# The transformative potential of artificial intelligence in solid organ transplantation

**DOI:** 10.3389/frtra.2024.1361491

**Published:** 2024-03-15

**Authors:** Mouhamad Al Moussawy, Zoe S. Lakkis, Zuhayr A. Ansari, Aravind R. Cherukuri, Khodor I. Abou-Daya

**Affiliations:** ^1^ Department of Surgery, Thomas E. Starzl Transplantation Institute, University of Pittsburgh, Pittsburgh, PA, United States; ^2^ Health Sciences Research Training Program, University of Pittsburgh, Pittsburgh, PA, United States

**Keywords:** artificial intelligence, machine learning, transplantation, prognostication, pre-evaluation, rejection diagnosis, monitoring

## Abstract

Solid organ transplantation confronts numerous challenges ranging from donor organ shortage to post-transplant complications. Here, we provide an overview of the latest attempts to address some of these challenges using artificial intelligence (AI). We delve into the application of machine learning in pretransplant evaluation, predicting transplant rejection, and post-operative patient outcomes. By providing a comprehensive overview of AI's current impact, this review aims to inform clinicians, researchers, and policy-makers about the transformative power of AI in enhancing solid organ transplantation and facilitating personalized medicine in transplant care.

## Introduction

Transplantation is the best treatment for end-stage organ failure. While significant advancements have led to improvement in access to transplantation and graft outcomes, challenges like organ shortage, chronic alloimmune injury, and post-transplant complications hinder reaching their full potential. Recent Artificial Intelligence (AI) breakthroughs have re-captured attention as they can address these challenges. AI, a multidisciplinary field encompassing machine learning (ML), deep learning, natural language processing, computer vision, and robotics, has rapidly advanced. Its application is facilitated by increased access to awesome computational power. Its utilization in transplantation has already yielded promising results, providing deeper insights into disease processes, and identifying potential prognostic factors. For example, ML algorithms can predict graft survival, optimize allocation, and guide immunosuppression, improving success rates. Additionally, AI-driven image analysis enhances organ quality assessment accuracy, efficiency, and diagnosis of rejection, guiding informed medical decisions. This scientific review comprehensively explores the use of AI in solid organ transplantation, examining its applications, current state of development, and future directions. We critically examine existing literature and evaluate AI's potential impact on pretransplant evaluation, rejection prediction, and post-transplant care. Ultimately, if properly employed, AI integration can potentially enhance patient outcomes.

## Pretransplant evaluation

Pretransplant evaluation is a rigorous process that involves multidisciplinary efforts (1). It includes donor assessment, organ evaluation, recipient risk stratification, and organ allocation. Currently, available methods to guide this process suffer from a lack of consistency, subjectivity, non-generalizability, and labor. AI can offer models to address these challenges, making the process more efficient and consistent. Evaluating donor kidneys for volume and structural abnormality is a key routine pre-transplant practice that usually depends on findings of donor Computed Tomography (CT) scans of the abdomen (2). Kidney volume is widely used as a surrogate to measure the nephron size (3). However, it does not differentiate between cortical and medullary compartments. One of the major drawbacks of such a practice is interobserver variability, the tedious work it requires, and its potential to misclassify a kidney as not transplantable erroneously. To unify kidney volume measurement, an automated kidney segmentation algorithm was developed and validated using contrast-enhanced CT exams from the Aging Kidney Anatomy study (4). The algorithm was trained on 1,238 exams and validated on 306 exams with manual segmentations. The Dice similarity coefficient, which measures the overlap between the automated and manual segmentations, was 0.94 for the cortex and 0.90 for the medulla, with a low percent difference bias. The algorithm was tested on 1,226 external datasets and performed on par with manual segmentation, with high Dice similarity metrics for both cortex and medulla segmentation. Along the same lines, Ram et al. explored the use of CT scans and supervised “dictionary learning” approach for screening donor lungs before transplantation (5). The algorithm was trained to detect pulmonary abnormalities on CT scans and predict post-transplant outcomes. The model was trained on a subset of 14 cases evenly split between accepted and declined donor lungs for transplantation. The training data consisted of CT scans of freshly procured human donor lungs, and the “ground truth” for training was determined by the final decision of 6 experienced senior thoracic surgeons. In other words, the model was trained to detect pulmonary abnormalities on CT scans by learning to associate specific CT image features with the classification of “accepted” or “declined” lungs, as determined by blinded thoracic surgeons. This algorithm assumes that each lung region on the CT scan can be accurately represented as a linear combination of very few dictionary elements. This allows the ML model to perform accurately, even with small data sets. The performance of the ML model was evaluated using a test set of 66 cases, consisting of 52 accepted and 14 declined donor lungs. The overall classification accuracy and area under the receiver operating characteristic curve (AUC) were 85% and 0.89, respectively.

On the other hand, lesion scores on procurement donor biopsies are commonly used to assess the health of deceased donor kidneys, guiding their utilization for transplantation (6). However, frozen sections present challenges for histological scoring, leading to inter- and intra-observer variability and inappropriate discard. Yi et al. constructed deep-learning-based models to assess kidney biopsies from deceased donors (7). The models, Mask R-CNN for compartment detection and U-net segmentation for tubule prediction enhancement, accurately identified tissue compartments and correlated with pathologists’ scores with the digital score for Sclerotic Glomeruli showing the strongest correlation (*R* = 0.75). In addition, Arterial Intimal Fibrosis % and Interstitial Space Abnormality %, also showed significant correlations with the corresponding pathologist scores (*R* = 0.43 and *R* = 0.32, respectively). The digital scores demonstrated higher sensitivity in identifying abnormalities in frozen biopsies, providing a more objective and accurate evaluation of features that were difficult to assess visually.

Another layer of preoperative assessment is identifying that transplantation is the suitable therapeutic approach to treat the patient's condition. This stands as a challenge in heart and lung transplantation, especially due to limited organ availability and the postoperative complications such invasive procedures carry. Therefore, identifying a suitable therapeutic approach for advanced heart failure patients is not an easy task, considering the absence of a consistent consensus. To address this issue, Yao et al. developed an interpretable ML algorithm to identify potential candidates for advanced heart failure therapies (8). The algorithm encoded observations of clinical variables into humanly understandable fuzzy concepts that match how humans perceive variables and their approximate logical relationships. Instead of using predefined thresholds, the method learned fuzzy membership functions during model training, allowing for a more nuanced representation of variables. The model included an algorithm for summarizing the most representative rules from single or multiple trained models. This summarization process helped provide a parsimonious set of rules that clinicians could easily interpret and use. When benchmarked against other interpretable models, the author's model achieved similar accuracy but better recall and precision and, hence, a better F1 score. The study had limitations such as a small dataset, limited follow-up data, and differences between the registry datasets regarding variable measurement and illness severity, which may affect the generalizability of the algorithm's findings. Although the overall performance of this model is not great, it represents a good starting point that can be improved by expanding the datasets of patients and providing a unified assessment of their risk factors.

Predicting graft function can be immensely helpful pre-transplant since resources can be allocated more efficiently to those who are more likely to benefit from the procedure. In fact, a ML prediction model for immediate graft function (IGF) after deceased donor kidney transplantation was developed (9). The model used well established algorithms such as eXtreme Gradient Boosting (XGBoost), light GBM and GBC. XGboost performed the best in predicting IGF and distinguishing it from delayed graft function (DGF) with an AUC of 0.78 and a negative predictive value (NPV) of 0.92. To keep in mind, XGBoost considers donor related factors such as age, diuresis, and KDRI. This suggests that creating a model for predicting IGF or DGF can enhance the selection of patients who would benefit from an expensive treatment, as in the case of machine perfusion preservation.

Prioritizing transplantation to patients who need it the most is essential in organ allocation. Indeed, organ transplantation might represent a life-saving option for a select group of patients (10). However, death while waiting for an organ is a crucial factor that is often overlooked (11). Recently, Bertsimas et al. developed and validated a prediction model called optimized prediction of mortality (OPOM) for liver transplant candidates (12). OPOM was generated based on available data within the Standard Transplant Analysis and Research (STAR) database, which are routinely collected on all waitlisted candidates. OPOM not only predicts a candidate's likelihood of dying but also becoming unsuitable for transplantation within three months. OPOM significantly improves its predictive capabilities compared to the current Model for End-Stage Liver Disease (MELD), especially within the sickest candidate population. OPOM maintained a significantly higher AUC values, allowing for a more accurate prediction of waitlist mortality. MELD's predictive capabilities deteriorated significantly with increasing disease severity, while OPOM's predictive power was maintained. This is important because the candidates with the highest disease severity warrant the most accurate mortality prediction for accurate prioritization on the liver transplant waitlist. MELD allocation includes using exception points for certain subpopulations of candidates, such as those with hepatocellular carcinoma (HCC). However, studies have shown a lack of survival benefits among patients undergoing liver transplantation based on MELD exception points (13). OPOM provides a more objective and accurate prediction of mortality without the need to raise MELD score artificially. OPOM uses more variables than MELD, including trajectories of change in lab values linked to MELD. These additional variables contribute to OPOM's accuracy in predicting mortality. Using OPOM could potentially save hundreds of lives each year and promote a more equitable allocation of livers.

Recipient evaluation is one of the most tedious pre-operative elements of transplantation (14). AI would provide more consistent and efficient models to help clinicians in this process. Physiological age that considers the cardiovascular fitness of transplant candidates is a well-established risk factor for postoperative complication and graft dysfunction. One study investigated the relationship between electrocardiogram (ECG) age, determined by AI, and mortality risk in kidney transplant candidates (15). The study found that ECG age was a risk factor for waitlist mortality. Determining ECG age through AI may help guide risk assessment in evaluating candidates for kidney transplantation. Patients with a larger age gap between ECG age and chronological age were more likely to have coronary artery disease and a higher comorbidity index. Adding the age gap to models that include chronological age improved mortality risk prediction by increasing the concordance of the models. The study demonstrated that each 10-year increase in age gap is associated with a more than 3-fold increase in mortality risk (HR 3.59 per 10-year increase; 95% CI: 2.06–5.72; *p* < 0.0001). Integrating the age gap in the model improved mortality risk prediction beyond chronological age and comorbidities in waitlisted patients.

Transplant candidate selection depends heavily on predicting post-transplant survival of each candidate (16). This allows for allocating scarce organs to patients who would benefit the most from organ transplantation. Such predictions are subjective and highly dependent on clinicians' judgment, institutional policies, patient and donor demographics, and post-transplantation time. A unified prediction model that accounts for all variables is needed. Yoon et al. introduced a personalized survival prediction model for cardiac transplantation called Trees of Predictors (ToPs) (17). The ToPs algorithm improves survival predictions in cardiac transplantation by providing personalized predictions for identified patient clusters and selecting the most appropriate predictive models for each cluster. Compared to existing clinical risk scoring methods and state-of-the-art ML methods, ToPs significantly improve survival predictions, both post- and pre-cardiac transplantation. Another model was developed for predicting kidney graft survival based on pretransplant variables using a Bayesian Belief Network (18). The model accurately predicted graft failure within the first year or within 3 years based on pretransplant donor and recipient variables. Variables such as recipient Body Mass Index (BMI), gender, race, and donor age were found to be influential predictors. The model was validated using an additional dataset and showed comparable results. The validation process showed that the predictive model had reasonable accuracy in identifying graft failure at 1 year and 3 years (AUC 0.59 and 0.60, respectively) after transplantation. Both studies can be used to optimize organ allocation by looking at different transplant outcomes: patient survival vs. graft survival. A model that can predict both might provide the utmost benefit in the allocation and listing process.

On the other hand, Brahmbhatt et al. evaluated different models, including the Lung Allocation Score (LAS), the Chan et al. 2019 model, a novel “clinician” model, and two ML models (Least Absolute Shrinkage and Selection Operator (LASSO) and Random Forest (RF)) for predicting 1-year and 3-year post-transplant survival (19). The LAS overestimated the risk of death, particularly for the sickest candidates. Adding donor variables to the models did not improve their predictive accuracy. The study highlights the need for improved predictive models in lung transplantation. The immediate post-transplant period represents a critical time point to consider when a candidate is evaluated for transplantation. To predict the 90-day survival of patients with acute-on-chronic liver failure (ACLF) following liver transplantation, Yang et al. explored the use of four ML classifiers: Support vector machine (SVM), logistic regression (LR), multilayer perceptron (MLP) and RF (20). Compared with conventional clinical scoring systems such as the MELD score, ML models performed better at predicting the 90-day post-transplant survival of ACLF patients. RF outperformed the rest of the employed models with an AUC of 0.949. Taken together, these findings suggest that ML models, especially RF classifier, can provide a better tool for organ allocation that is tailored to clinical patients' outcomes. In the pediatric population, Garcia-Canadialla et al. explored the use of ML to analyze echocardiographic and clinical data in pediatric patients with dilated cardiomyopathy (DCM) (21). k-means identified groups with similar phenotypes, allowing for the identification of 5 clinically distinct groups associated with differing proportions of death or heart transplant (DoT). The study identifies distinct patient groups with different clinical characteristics and outcomes and suggests that ML can improve prognostication and treatment of pediatric DCM. The integration of comprehensive echo and clinical data helps identify subgroups of DCM patients with varying degrees of dysfunction, aiding in risk stratification and providing insights into the pathophysiology of pediatric heart failure.

Last, an important area in transplantation involves HLA matching. Neimann et al. developed a deep learning-based algorithm called Snowflake (22), which considers allele-specific surface accessibility in HLA matching. The focus of Snowflake is to calculate solvent accessibility of HLA Class I proteins for deposited HLA crystal structures, supplemented by constructed HLA structures through the AlphaFold protein folding predictor and peptide binding predictions of the APE-Gen docking framework. This allows for the refinement of HLA B-cell epitope prediction by considering allele-specific surface accessibility. This can improve the accuracy of B-cell epitope matching in the context of transplantation. This approach may also help in identifying potential gaps in epitope definitions and verifying Eplets with low population frequency by antibody reaction patterns. It remains to be shown whether this approach benefits outcomes. Table 1 summarizes all studies reviewed in this section.

**Table 1:** Summary of studies involving AI in pre-transplant evaluation.

Authors	Year	Application	Test	Comparison	Performance	Limitations	Data availability
Korfiatis et al. (4)	2022	Measuring kidney, cortex and medulla volumes	Segmentation using modified 3D U-Net architecture	Manual segmentation using ITK-SNAP	– Dice similarity metric of 0.94 (R and L cortex), and 0.9 (R and L medulla) – Similar interobserver variability	– Lack of generalizability due to small sample size and lack of training data samples from multiple centers. – Limited knowledge in population with less healthy or diseased kidneys	The Aging Kidney Anatomy Study: pre-donation scans from 2012 to 2015 from Mayo Clinic Arizona and Minnesota (training), and Cleveland Clinic (external validation and testing).
Ram et al. (5)	2023	Evaluating donor lung for any abnormalities prior to transplantation	Dictionary learning algorithm based on ex-situ lung CT scan	Clinical judgement of blinded 6 experienced thoracic surgeons		– Lack of generalizability due to the small sample size and the single center nature of the trial.	100 subjects enrolled as part of a single-center prospective trial from 2016 to 2018 from the University Hospital Leuven
Yi et al. (7)	2022	Lesion score for donor kidney biopsy pre-transplantation	Deep learning based tissue compartment recognition and whole slide image investigation	Pathologist assessment	– Digital score paralleled pathologist score with the strongest correlation for glomerular sclerosis and lowest for Interstitial Space Abnormality – Superior association with post-transplant estimated glomerular filtration rates and graft loss	– Low performance in detecting ISA is due to challenges in reading tubular findings on frozen biopsies. – Lack of generalizability	OPTN cohort
Yao et al. (8)	2022	Identifying patients with advance heart failure that require heart transplantation and durable LVAD implantation	Machine learning model based on principles of tropical geometry and fuzzy logic	Other machine learning models	– F1 score of 43.8%, recall of 51.1, and precision of 46.9% – Outperform Decision Tree and Fuzzy Inference Classifier in their transparency and ability to provide a parsimonious set of rules	– Samples used to traing the model does not reflect the real-world setting in terms of advanced therapies delivery – Model identifies patients who require advanced heart therapy but does not identify patients who benefit the most of such therapies.	REVIVAL (Registry Evaluation of Vital Information for VADs in Ambulatory Life) and INTERMACS (Interagency Registry for Mechanically Assisted Circulatory Support) registries
Quinino et al. (9)	2023	Prediction for immediate graft function in deceased kidney allograft recipients	Popular Machine Learning algorithms: XG Boost,Light Gradient Boosting Machine, Gradient Boosting VClassifdier, Logistic Regression,CatBoost Classifier, AdaBoost classifier, and Random Forest classifier.		XGBoost outperforms the other models: – AUC of 0.78 (CI:0.71–0.84) – Sensitivity of 0.64 – Specificity of 0.78	– Homogeneity of the sample used for training and testing of various models. – Small sample size	Patients undergoing their first deceased donors KTx at our Transplant Service between January 1, 2010, and December 31, 2019
Bertsimas et al. (12)	2019	Evaluating liver candidate's 3-month waitlist mortality or removal	Optimal classification tree machine learning model (OPOM)	Model for End-Stage Liver Disease (MELD)	– OPOM outperformed MELD with an AUC of 0.859 Predictive power of OPOM is preserved at high disease severity, while MELD performance deteriorates with disease severity.	– Generalizability concerns especially since the model was trained and tested on a single center dataset	Organ Procurement and Transplantation Network (OPTN) Standard Transplant Analysis and Research (STAR) dataset
Lorenz et al. (15)	2023	– Evaluating the ECG age (physiological age) – Integrating ECG age into predictive model of pre-transplant waitlist mortality.	Convolutional neural network	Chronological age	Each 10-year increase in the age gap is associated with a more than 3-fold increase in mortality risk	Inability to associate the elevated age gap with a specific ECG finding. Inaccuracies in the cardiac risk factors listed in training dataset Selection biases in the training population	All adult patients who underwent evaluation for KT alone or simultaneous pancreas/kidney transplant at Mayo Clinic in Minnesota between December 2014 and December 2019
Yoon et al. (17)	2018	Risk scoring for heart transplant candidates’ stratification	Trees of Predictors (ToPs)	Clinical risk scoring model: Donor Risk Index, Risk-Stivity of stratification Score, and the Index for Mortality Prediction After Cardiac Transplantation	ToPs outperforms the best clinical risk scoring model (RSS) with an AUC of 0.66 vs. 0.587 and a sensitivity and specificity of 80%.	Model fails to provide pre-transplantation risk assessment	The United Network for Organ Sharing (UNOS) database
Brown et al. (18)	2012	Predicting kidney graft survival	Bayesian Belief Network using pretransplant donor and recipient variables		Predictive power for graft failure with the first year or within 3 years with an AUC of 0.63 and sensitivity of 40% and specificity of 60%.	Information loss due to binning continuous data	The United States Renal Data System (USRDS)
Brahmbhatt et al. (19)	2022	Predicting 1 year mortality based on the Lung Allocation Score (LAS)	Lung Allocation Score (LAS)	Chan et al, 2019 model Random Forest Clinician model LASSO	Low area under the curve of all models (0.55–0.62) Reasonable NPVs (0.87–0.9) Poor PPV of less than 0.25	Limited variables available that might compromise the model predictive accuracy Fewer outcome data on recent transplanted patients LAS overestimate the risk of death limting access to transplantation for certain patients	The United Network for Organ Sharing database (UNOS)
Yang et al. (20)	2022	Predicting 3 month survival of acute on chronic liver failure patients post liver transplantation	Four machine learning models: support vector machine, logistic regression, multilayer perceptron, random forest	Five conventional score systems: MELD score, ABIC, CLIF-SOFAs and CLI-C ACLFs	ML models outperformed conventional models in predicting short term survival of AOCLF patients post transplantation. RF performed the best of all models with an AUC of 0.94, meanwhile the best performing conventional model was MELD with an AUC of 0.704	Single center Retrospective data Lack of donor data Small sample size	132 ACLF patients undergoing LT were enrolled from the Transplantation Center, Third Xiangya Hospital, Central South University between March, 2012 and December, 2019
Garcia-Canadilla et al. (21)	2022	Identifying the risk factors for death or heart transplantation in pediatric patients with dilated cardiomyopathy	Machine learning model		Predict patients that are at risk of ToD Cluster at risk patients into different pheno-groups	Small sample size Omission of clinically relevant data during training that include biomarkers and genotypes	Data from children, 0–18 years of age, diagnosed with idiopathic, familial, or genetic DCM, presenting to our institution between 6/2004 (when digital echocardiography started) and 2016

## AI and diagnosis of rejection

The Banff Automation System is an automated histological classification system for diagnosing kidney transplant rejection (23). This tool has been developed to eliminate or minimize misclassification due to the misinterpretation of the histological findings of pediatric and adult kidney allograft biopsies that result from the complex and tedious international Banff classification rules. The Banff Automation System was developed to improve the precision and standardization of rejection diagnosis in kidney transplantation by providing a comprehensive and automated algorithm based on the Banff classification system. The system has been tested on kidney transplant recipients and has shown promising results in reclassifying rejection diagnoses and improving patient risk stratification. In a user-friendly fashion, this system allows clinicians to input all the necessary parameters for an accurate Banff evaluation, including histological lesion scores, relevant associated histological lesions, non-rejection-related associated diagnoses, C4d staining, circulating anti-HLA DSA, electron microscopy results, and molecular markers if available. The system computes the inputs and generates a diagnosis according to the Banff classification, along with notes and suggestions for complex cases. It also provides a decision-tree visualization to help users understand the process that led to the diagnosis. In fact, the cases that were reclassified correctly as rejection by the system after being diagnosed as rejection had worse graft survival (HR = 6.4, 95% CI: 3.9–10.6, *p* value < 0.0001). In contrast, the cases that were initially diagnosed as rejection by pathologists and then reclassified as no rejection by the system had better graft survival (HR = 0.9, 95% CI: 0.1–6.7, *p* value = 0.941). By automating the diagnostic process and providing standardized criteria, the Banff Automation System reduces inter-observer variability and improves the consistency of rejection diagnosis. It ensures that clinicians follow the established Banff rules and guidelines, leading to more precise and reliable diagnoses. The system has been validated by independent nephropathologists and transplant physicians in real-life clinical use, further confirming its accuracy and consistency.

Another group that has developed a ML model to help pathologists provide a more accurate diagnosis of kidney allograft rejection is Zhang lab (24). The group trained their pipeline using PAS-stained biopsies based on two deep learning structures: the masked region-based convolution neural network which help differentiate between various kidney compartments and detect mononuclear leukocytes, and U-Net that allowed tissue segmentation. The developed pipeline was used to investigate clinically whole slide images (WSI) in order to correlate them with an allograft outcome. Two scoring systems were developed: one for initial biopsies (ITAS) and another for post-transplant biopsies (Composite Damage Score). Both scores strongly predicted graft loss within a year, acting as early warning signs. This superior accuracy compared to Banff scoring system suggests deep learning could become a more reliable and objective tool for assessing transplant health. By stratifying patients based on their individual graft loss risk, personalized treatment plans and closer monitoring for high-risk patients become possible.

Along the same lines, recent work has focused on better describing the severity of rejection to complement a rejection diagnosis since rejection severity dictates clinical management. Naesens’ group developed and validated a tool to describe the chronicity and severity of chronic alloimmune injury in kidney transplant biopsies (25). They used a semi-supervised consensus clustering algorithm called RejectClass to identify four chronic phenotypes based on the chronic Banff lesion scores that are associated with graft outcomes. The total chronicity score which represents the sum of four weighted chronic lesion scores is strongly correlated with graft failure, independent of acute lesion scores. On the other hand, Labriffe et al. trained a boosted decision tree model that performed well in diagnosing distinct types of rejection and fibrosis in kidney graft biopsies (26). The antibody-mediated rejection (ABMR) classifier yielded AUC of 0.97, 0.97, and 0.95, and precision recall (PR) area under the curve of 0.92, 0.72, and 0.84 for the MH Hannover, KU Leuven, and Necker Paris datasets, respectively. The T-cell-mediated rejection (TCMR) model had AUCs of 0.94, 0.94, and 0.91, and PR area under the curve of 0.91, 0.83, and 0.55 for the same datasets. IFTA classifier had AUC ≥0.95 for both ROC and PR curves in all datasets. This model helps standardize histopathological rejection diagnosis, eliminating outcome uncertainty in guiding clinical management or clinical trials. However, this model depends on the biopsy procedure, sampling, preparation, and elementary lesion grading, which are all operator and observer dependent. This model assumes that all biopsies are performed, processed, and read accurately. Such an assumption might limit this model's accuracy and reproducibility.

Although histological classification is the gold standard for graft rejection diagnosis, this method suffers from inconsistencies and lack of reproducibility due to the complexity of the Banff classification system. Efforts have been made recently to use AI to integrate transcriptomic or proteomic data to establish diagnostic models (27). For instance, Fang et al. used quantitative label-free mass spectrometry analysis on formalin-fixed, paraffin-embedded biopsies from kidney transplant patients to identify proteomic biomarkers for T-cell-mediated kidney rejection (28). The label-free proteomics method allowed for quantifying 800–1,350 proteins per sample with high confidence. Differential expression analysis was then conducted to identify differentially expressed proteins (DEPs) that could serve as potential biomarkers for TCMR. Three different ML algorithms, including LDA, SVM, and RF, were applied to the protein classifiers derived from the DEPs identified in the label-free proteomics analysis. The group validated the diagnostic model for rejection using an independent sample set consisting of 5 TCMR and five stable allograft (STA) biopsies. The leave-one-out cross-validation result demonstrated that the RF-based model achieved the best predictive power over two other ML models. In a follow-up blind test using an independent sample set, the RF-based model yields 80% accuracy for TCMR and 100% for STA. When applying the established RF-based model to two public transcriptome datasets, 78.1%–82.9% sensitivity and 58.7%–64.4% specificity was achieved.

Van Baardwijik used genes from the Banff-Human Organ Transplant (B-HOT) panel to develop a decentralized molecular diagnostic tool for kidney transplant biopsies (29). They developed a RF classifier and compared the performance of the B-HOT panel with other available models. The B-HOT+ model, which included the B-HOT panel genes plus six additional genes, achieved high accuracy in classifying rejection types. It achieved an AUC of 0.965 for non-rejection (NR), 0.982 for ABMR, however, lacked the sample size to test AUC for TCMR during cross-validation within the GSE98320 dataset. In the independent validation within the GSE129166 dataset, the B-HOT+ model achieved an AUC of 0.980 for NR, 0.976 for ABMR, and 0.994 for TCMR. The B-HOT+ model had higher AUC scores than other models, demonstrating its superior performance in classifying kidney transplant biopsy Banff categories.

Some groups went above and beyond to create a new classification system based on molecular phenotyping, claiming that it is superior to histological assessment in diagnosing rejection due to the subjectivity of the latter method. The Molecular Microscope Diagnostic System (MMDx)-Kidney study group presented a new system for assessing rejection-related disease in kidney transplant biopsies using molecular phenotypes (30). The researchers collected microarray data from over 1,200 biopsies. They used supervised ML methods (linear discriminant analysis, regularized discriminant analysis, mixture discriminant analysis, flexible discriminant analysis, gradient boosting machine, radial support vector machine, linear support vector machine, RF, neural networks, Bayes glm, and generalized linear model elastic-net) to generate classifier scores for six diagnoses: no rejection, TCMR, early-stage ABMR, fully developed ABMR, late stage ABMR, and Mixed ABMR and TCMR. They then used unsupervised archetypal analysis to identify six archetype clusters representing different rejection-related states. The study found that the molecular classification provided more accurate predictions of graft survival than histologic diagnoses or archetype clusters.

The use of AI to improve rejection diagnoses has been expanded to involve different organ transplantation models. The use of RNA sequencing (RNA-seq) and a RF-based classifier to improve the diagnosis of acute rejection (AR) in heart transplant recipients stands as an example (31). Peining et al. performed RNA-seq analysis on endomyocardial biopsy samples from 26 patients and identified transcriptional changes associated with rejection events that were not detected using traditional histopathology. They developed a diagnostic and prognostic signature that accurately predicted whether the next biopsy would show rejection with 90% accuracy. A weighted long-term memory (LSTM) model was developed in liver transplantation to detect graft fibrosis (32). The model outperformed [AUC = 0.798 (95% CI: 0.790 to 0.810)] multiple other conventional ML algorithms and serum fibrosis biomarkers. The weighted LSTM model showed promising results in improving the early diagnosis of graft fibrosis after liver transplantation.

Halloran et al. focused on developing molecular assessments for lung transplant biopsies and evaluating the impact of molecular TCMR on graft survival (33). The researchers used microarrays and ML to assign TCMR scores to transbronchial biopsies and mucosal biopsies. The researchers assessed molecular TCMR in lung transplant biopsies using the MMDx, which is a validated system combining microarray-based measurements with ML-derived algorithms. This system was adapted for the INTERLUNG study to define the molecular phenotype of lung rejection. Molecular TCMR was associated with graft loss, while histologic rejection and donor-specific antibodies were not. The study suggests that molecular TCMR can predict future graft failure in lung transplant recipients. Table 2 summarizes all studies reviewed in this section.

**Table 2:** Summary of studies involving AI in rejection diagnosis.

Authors	Year	Application	Test	Comparison	Performance	Limitations	Data availability
Yoo et al. (23)	2023	Evaluating kidney biopsy for diagnosis of rejection	Banff automation System	International Banff classification by pathologists	In adults: Antibody mediated rejection reclassification rate of 29.75% T cell mediated rejection reclassification rate of 54.29% Rejection reclassification rate of 7.32% In pediatrics: ABMR reclassification rate of: 30.77% TCMR rate of:30.77%	The automated system doesn’t change the pathologist's grading and scoring of a specific lesion. The classification doesn’t consider the clinical history of the patient Classification might require changes and updating in case Banff classification system changes	4,409 kidney transplant biopsies from three prospective multicentric cohorts of adult and pediatric kidney transplant recipients (Paris Transplant Group cohort, University of Wisconsin-Madison's cohort and the international pediatric cohort NIH project 1R21DK122229-01) and two large clinical trials (KTD-Innov NCT03582436 and EU-TRAIN NCT03652402)
Zhang et al. (24)	2022	Diagnosing Kidney allograft rejection based on whole slide image. Providing a prognostic assessment based on biopsy finding	Deep leaning models: mask Region-based Convolution Neural Network And *U*-Net	Banff scoring by pathologists	The deep learning model effectively identified and measured various pathological lesions within the biopsies, outperforming Both ITAS and the Composite Damage Score were highly predictive of graft loss within one year for baseline and post-transplant biopsies, respectively.	Small sample size Single center retrospective data The model doesn’t accurately microvascular inflammation and arteritis The model was not trained to detect and diagnose acute cellular rejection	The Genomics of Chronic Allograft Rejection (GoCAR) Australian Chronic Allograft Dysfunction (AUSCAD)
Vaulet et al. (25)	2022	Assessment of kidney lesion chronicity and severity	Semisupervised consensus clustering algorithm, RejectClass		Hazard ratio of 5.01 (CI: 2.83–7; *p*-vaalue <0.001) independent of the total activity	Algorithm performance depends heavily on the histological Banff scoring which is subject to inter- and intra- observer variability	All kidney transplantations performed at the University Hospitals Leuven between March 2004 and February 2013 (training) Electronic databases of Lyon University Hospitals and the Necker Hospital in Paris (validation)
Labriffe et al. (26)	2022	Diagnosing rejection based on integrated clinical and histological findings	XGBoost Classifier		High AUC of 0.95–0.97 for ABMR High AUC of 0.91–0.94 of TCMR High AUC > 0.96 for IFTA. 95% accuracy in discriminating active vs. chronic ABMR.	The approach depends heavily on biopsy procedure, and elementary lesion pathology evaluation.	BIOMArkers of Renal Graft INjuries (BIOMARGIN, NCT02832661) and Reclassification using OmiCs integration in KidnEy Transplantation (ROCKET, funded by ERACoSysMed) (training) Data from three transplant centers: KU Leuven (Belgium), MH Hannover (Germany), and Necker Paris (France) (validations)
Fang et al. (28)	2023	Providing a more efficient, accurate and reproducible rejection diagnosis	Machine learning models-three algorithms: LDA,SVM, and RF to provide a proteomic based TCMR diagnosis	Histological evaluation	RF classifier outperformed the other two ML algorithms RF based model achieved an accuracy of 80% for TCMR and 100% for stable RF-based model sensitivity of 78.1–82.9 and specificity of 58.7–64.4	Very small cohort Identified molecular marker by proteomic technology need to be validated and verified before being used for diagnostic purposes	Kidney transplantation cohort from the University of Pittsburgh Medical center
Baardwijk et al. (29)	2022	Providing more accurate, efficient and reproducible molecular diagnosis of kidney rejection	Multilabel random forest model trained on B-Hot panel genes and Feature Selection model most predictive genes	Histological evaluation of kidney biopsy	Mean accuracy of 92.1% AUC of 0.965 for non-rejection and 0.982 for ABMR	Retrospective data Interobserver variability in evaluating kidney biopsy pathology. No TCMR data	Two public gene expression datasets (GSE98320 and GSE129166) from the Gene Expression Omnibus database
Reeve et al. (30)	2017	Enhancing rejection diagnosis	Supervised machine learning methods: archetypal analysis	Histological evaluation of kidney biopsy	54% molecular discrepancy with histological rejection diagnoses 16% showed molecular rejection despite no histological rejection	Retrospective data Molecular elements might require validation prior to use	Gene Expression Omnibus (GEO) database
Piening et al. (31)	2022	Improving the sensitivity and specificity of histopathological evaluation of endomyocardial biopsy for assessment of cardiac graft rejection	Differential expression and RF based machine learning model	Histopathological evaluation of endomyocardial biopsy	A prognostic signature of non-histological rejection, predicting with a 90% accuracy histopathological determined rejection on subsequent biopsies.	Small sample size	CTOT-03 study (https://clinicaltrials.gov/study/NCT00531921)
Azhie et al. (32)	2023	Early detection of fibrosis in liver allografts	Weighted long short-term memory	– Liver biopsy (gold standard) LSTM, recurrent neural network, tamporal conventional network, RF, SVM,LR,Lasso regression, Ridge regression, clinical findings (Aspartate aminotransferase to platelet ratio index, fibrosis-4 index and transient elastography)	Weighted LSTM outperformed other available model in predicting advanced liver fibrosis with an AUC of 0.798	Small biopsy sample size Variability In liver biopsy procedure and interpretation Models are trained on patients whose clinical state warranted a biopsy, making the extrapolation of this model to include follow up-outpatient settings questionable Time consuming method	Adults who had received a liver transplantation (aged ≥18 years at transplant) with at least one post-transplant liver biopsy done at the University Health Network (UHN), Toronto, ON, Canada, between Jan 1, 1992, and June 30, 2020
Halloran et al. (33)	2020	Developing a molecular assessment for transbronchial lung biopsy and mucosal biopsies and establishing the impact of molecular TCMR on graft survival	Unsupervised machine learning techniques: Archetypal analysis (AA) and principal component analysis (PCA)	Histological evaluation of bronchial and mucosal biopsies	TCMR scores in transbronchial biopsies match those in mucosal biopsy.	Lack of centralized histology and DSA assessment of the biopsies Kaplan Meier survival analyses were not adjusted to confounding variables RF of graft loss omitted important variables such as donor age, and primary graft dysfunction CLAD status needs reassessments in the light of new CLAD consensus definition.	Adult lung transplant recipients from 10 centers

## AI and immunosuppression in transplantation

Immunosuppression is the mainstay postoperative measure to maintain graft tolerance and prevent graft loss (34). However, dosing of immunosuppressive drugs requires iterative monitoring to reach a therapeutic blood level, especially during the immediate postoperative period and during stresses such as infections or rejection episodes. This process requires frequent blood draws and office visits. AI can offer a non-invasive model to predict immunosuppressive drug blood levels. One of the earliest AI-based studies in transplantation utilized evolutionary algorithms (EAs), symbolic AI, to create a pharmacokinetic model for predicting cyclosporine blood levels in 101 heart transplant patients (35). The EA-based software tool showed accurate predictions and a strong correlation with observed levels. The mean percentage error between the predicted and observed cyclosporine concentrations for the training data set was 7.1% (0.1%–26.7%), and for the test data set, it was 8.0% (0.8%–28.8%). The correlation coefficient between predicted and observed cyclosporine levels was 0.93 for the training data set and 0.85 for the test data set. Besides using evolutionary algorithms, other AI techniques that have been trained in predicting drug levels and outcomes in transplant patients include neural networks. For example, a study by Chen et al. used a neural network with a genetic algorithm to predict tacrolimus blood levels in 32 liver transplantation patients (36). The neural network prediction for tacrolimus blood levels was not significantly different from the observed value by a paired *t*-test comparison (12.05 ± 2.67 ng/ml vs. 12.14 ± 2.64 ng/ml, *p* = 0.80). More recent work by Min et al. focusing on 777 pediatric transplant recipients utilized GWAS by SNP arrays to pinpoint 14 SNPs independently associated with tacrolimus levels. Linear regression with stepwise backward deletion vs. LASSO model to determine the significant predictors of log-transformed dose-adjusted T1 levels (37). Both traditional and ML approaches selected organ type, age at transplant, rs776746, rs12333983, and rs12957142 SNPs as the top predictor variables for dose-adjusted 36- to 48-h post tacrolimus initiation (T1) levels. In another study, an ML model outperformed traditional Bayesian estimation methods and showed potential for routine tacrolimus exposure estimation and dose adjustment (38). Xgboost model yielded excellent AUC estimation performance in test datasets, with a relative bias of less than 5% and a relative root mean square error (RMSE) of less than 10%. Additionally, the ML models outperformed the MAP-BE method in six independent full-pharmacokinetic datasets from renal, liver, and heart transplant patients. These findings suggest that the ML models can provide more accurate estimation of tacrolimus interdose AUC and can be used for routine tacrolimus exposure estimation and dose adjustment. Table 3 summarizes all studies reviewed in this section.

**Table 3:** Summary of studies involving AI in immunosuppression monitoring.

Authors	Year	Application	Test	Comparison	Performance	Limitations	Data set	Data availability
Hoda et al. (35)	2005	Prediction of cyclosporine levels in heart transplantation patients	EVIS-Evolutionary algorithms	Blood levels of cyclosporine (CyA)	AUC ranging between 80 and 90% Correlation coefficient between predicted and observed CyA levels for tested data set was 0.85 (*p* -value <0.001)	Algorithm doesn’t account for patients’ lifestyle and non-compliance. Small sample size Chen	101 randomly selected adult heart transplant patients from the Heart and Lung Transplantation Center, University of Vienna Medical School	
Chen et al. (36)	1999	Prediction of tacrolimus levels in liver transplantation patients	Neural Network combined with genetic algorithm	Blood levels of tacrolimus	Average difference between predicted and observed tacrolimus levels is 1.74 ng/ml	Small sample size Retrospective nature of the training data set	Liver transplant patients from the University of Iowa Hospitals and Clinics	
Min et al. (37)	2022	Prediction of tacrolimus level in pediatric solid organ transplant recipients	Linear regression using clinical predictors, genetic variables and a combination of both	Blood levels of tacrolimus	The combined prediction model has the lowest prediction error and explains 30% of the variation in dose-adjusted tacrolimus levels.	Only applies for pediatric population Selection bias in the populations (underrepresentation of African Americans or other racial and ethnic groups)	POSITIVE cohort	
Woillard et al. (38)	2021	Prediction of tacrolimus levels in transplant patients	XgBoost machine learning model	Blood levels of tacrolimus	Accurate estimation of tacrolimus interdose AUC	Small sample size Retrospective nature of the study	TAC AUC estimation and dose recommendation cohort	

## Prognostication

Transplant patients require close follow-up post-operatively to monitor graft function and detect any rejection episodes (39). Providing clinicians with a tool to predict rejection or graft failure would help patients and improve their outcomes. For instance, Mulugeta et al. developed a model to predict the risk of graft failure in kidney transplant recipients (40). Analyzing data from a retrospective cohort of kidney transplant recipients at the Ethiopian National Kidney Transplantation Center, the study employed various ML algorithms to address the imbalanced nature of the data, characterized by a disproportionate representation of successful transplants compared to graft failures. The authors employed a combination of strategies, including hyperparameter tuning, probability threshold moving, tree-based ensemble learning, stacking ensemble learning, and probability calibrations. These techniques aimed to optimize the performance of individual ML models, adjust classification thresholds to account for the imbalanced data, harness the collective strength of multiple tree-based models, leverage stacking ensemble learning to create a more robust prediction system, and ensure the predicted probabilities align with the actual graft failure rates. The study's findings demonstrated the efficacy of ML algorithms in predicting renal graft failure risk, particularly when employing ensemble learning methods like stacking ensemble learning with stochastic gradient boosting as a meta-learner. This approach achieved an AUC-ROC of 0.88 and a brier score of 0.048, indicating high discrimination and calibration performance. Additionally, feature importance analysis revealed that chronic rejection, blood urea nitrogen, and creatinine were the most significant predictors of graft failure. This study highlights the potential of ML models as valuable tools for risk stratification and personalized care in renal transplantation.

In addition, Tian et al. explored the development and evaluation of a ML-based prognostic model to predict survival outcomes for patients following lung transplantation (41). Utilizing data from the United Network for Organ Sharing (UNOS), the authors developed a Random Survival Forest (RSF) model, incorporating a comprehensive set of clinical variables. The RSF model demonstrated superior performance compared to the traditional Cox regression model, achieving an AUC of 0.72 and a C-index of 0.70. Stratification of patients based on the RSF model's predictions revealed significant differences in survival outcomes, with a mean overall survival of 52.91 months for the low-risk group and 14.83 months for the high-risk group. Additionally, the RSF model proved effective in predicting short-term survival at one month, with a sensitivity of 86.1%, a specificity of 68.7%, and an accuracy of 72.9%. These findings suggest that the RSF model holds promise as a valuable tool for risk stratification and personalized care management for lung transplant recipients, enabling clinicians to make informed decisions regarding treatment strategies and resource allocation.

Not only is survival an issue but complications can arise after transplantation. Chen et al. used various preoperative and intraoperative factors to predict the occurrence of postoperative sepsis (42). Their RF Classifier model outperformed the commonly used Sequential Organ Failure Assessment (SOFA) score in predicting postoperative sepsis in liver transplant patients. The model demonstrated a higher AUC in the validation set than the SOFA score (0.745 vs. 0.637).

Additionally, Banerjee et al. developed and evaluated a ML-based survival tree algorithm, named ReSOLT (Recipient Survival After Orthotopic Liver Transplantation), to predict post-transplant survival outcomes for liver transplant recipients. The authors used the UNOS transplant database (43). They identified eight significant factors that influence recipient survival: recipient age, donor age, recipient primary payment, recipient hepatitis C status, recipient diabetes, recipient functional status at registration and at transplantation, and deceased donor pulmonary infection. The ReSOLT algorithm effectively stratified patients into 20 subgroups with distinct survival probabilities, demonstrated by significant log-rank pairwise comparisons (*p* < 0.001). The estimated 5- and 10-year survival probabilities varied considerably among the subgroups, highlighting the algorithm's ability to provide patient-specific survival estimates. Furthermore, the ReSOLT algorithm demonstrated superior performance compared to traditional survival analysis methods, achieving an AUC of 0.742 and a C-index of 0.728. These findings suggest that the ReSOLT algorithm can serve as a valuable tool for personalized risk stratification and treatment planning in liver transplant recipients, enabling clinicians to make informed decisions that optimize patient outcomes.

Moreover, Ivanics et al. investigated the feasibility and effectiveness of ML-based 90-day post liver transplant mortality prediction models using national liver transplantation registries from Canada, United States of America, and United Kingdom (44). The models exhibited satisfactory performance within their respective countries, but their applicability across different countries was restricted. The average performance metrics across countries were AUC-ROC of 0.70, accuracy of 0.74, sensitivity of 0.76, and specificity of 0.67. These findings indicate that ML-based mortality prediction models could be beneficial for risk stratification and resource allocation within individual countries, but caution is advised when applying them across diverse healthcare systems and patient populations.

Along the same lines, the Paris Transplant Group evaluated the efficacy of ML algorithms in predicting kidney allograft failure compared to traditional statistical modeling approaches (45). Utilizing data from a large retrospective cohort of kidney transplant recipients from 14 centers worldwide, Truchot et al. compared the performance of various ML models, including logistic regression, naive Bayes, artificial neural networks, RF, bagged tree, and stochastic gradient boosting, against a Cox proportional hazards model. The results demonstrated that no ML model outperformed the Cox model in predicting kidney allograft failure. The Cox model achieved an AUC of 0.78, while the best-performing ML model, stochastic gradient boosting, achieved an AUC of 0.76. These findings should not discourage the application of ML in predicting graft outcomes; however, scientists should think more creatively about how to best utilize ML in hopes of outperforming current methods. One aspect of ML techniques that outshines statistical modeling is the clustering and/or projection of unique patient groups. For instance, Jadlowiec et al. employed an unsupervised ML approach on the UNOS OPTN database to categorize distinct clinical phenotypes among kidney transplant recipients experiencing DGF (46). The group applied consensus clustering techniques to identify four distinct clusters of DGF patients, each characterized by unique clinical profiles and associated outcomes. These clusters exhibited varying levels of severity, with one cluster associated with a significantly higher risk of allograft failure and death compared to the others. The findings underscore the heterogeneity of DGF and highlight the potential of ML in identifying subgroups of patients with distinct clinical trajectories and risk profiles, enabling personalized treatment strategies and improved patient outcomes. Similarly, our group has utilized t-SNE, a dimensionality reduction technique, to uncover strata of patients suffering from borderline rejection (47). We detected 5 distinct groups of Borderline rejection patients with markedly different outcomes.

Length of stay after transplantation reflects postoperative complications. Identifying the patients at risk is essential to provide them with the most optimal care to carry them safely beyond the critical post-operative period. Gupta et al. investigated the factors associated with prolonged hospital length of stay (PLOS) after pediatric heart transplantation and developed a predictive model using ML and logistic regression techniques (48). Analyzing data from the Pediatric Heart Transplant Society database, the authors identified several factors associated with PLOS, including younger age, smaller body size, congenital heart disease, preoperative use of extracorporeal membrane oxygenation, and longer cardiopulmonary bypass time. The ML model, which incorporated 13 variables, demonstrated superior performance in predicting PLOS compared to the logistic regression model. This suggests that ML approaches may provide a useful tool for identifying patients at risk of PLOS and optimizing post-transplant care strategies.

Outcome prediction for organ transplantation is not only critical for organ allocation, patient stratification but also post-operative patient risk assessment. Due to the iterative list of variables that come into play, ML provides a user friendly and more efficient model to compute outcome prediction. Miller et al. found that ML and statistical models can be used to predict mortality post-transplant, with RF achieving the highest AUC for predicting 1-year survival at 0.893 (49). However, the overall prediction performance was limited, especially when using the rolling cross-validation approach. Additionally, there was a trend toward higher prediction performance in pediatric patients. In predicting mortality post-transplant, the performance of tree-based models, such as XGBoost and RF, was substantially higher compared to linear regression models. In the shuffled 10-fold cross-validation, RF achieved the highest AUC for predicting 1-year survival at 0.893, followed by XGBoost with an AUC of 0.820. In contrast, linear regression exhibited much lower prediction performance, with logistic regression achieving an AUC of 0.641 in the rolling cross-validation procedure.

Another study by Nitski et al. utilized deep learning algorithms to predict complications resulting in death after liver transplantation (50). The algorithms outperformed logistic regression models, with the Transformer model achieving the highest accuracy. The models were trained and validated using longitudinal data from two prospective cohorts. They predicted various outcomes such as graft failure, infection, and cardiovascular events and identified important variables for prediction. The study highlights the potential of ML in improving post-transplant care. Deep learning algorithms outperformed logistic regression models in predicting complications resulting in death after liver transplantation. The AUC for the top-performing deep learning model was 0.807 for 1-year predictions and 0.722 for 5-year predictions, while the AUC for logistic regression models was lower. The deep learning models achieved the highest AUCs in both datasets, indicating their superior performance in predicting long-term outcomes after liver transplantation.

AI can offer prediction models for graft function or rejection post-transplantation. Tongprayoon et al. used an unsupervised ML consensus clustering approach to stratify black kidney transplant recipients based on their baseline risk factors, donor profile index score, and allograft type (deceased vs. living donor) (51). The authors found clinically meaningful differences in recipient characteristics among the four clusters identified based on DGF. However, after accounting for death and return to dialysis, there were no significant differences in death-censored graft loss between the clusters. This suggests that recipient comorbidities, rather than DGF alone, play a key role in determining survival outcomes. Other factors such as immunologic, cardiac, metabolic, and socioeconomic contributors may also contribute to the varying outcomes associated with DGF. Overall, the study highlights the need to consider recipient characteristics and comorbidities when evaluating the impact of DGF on graft survival. Such a model offers a tool to clinicians to better assess their transplant candidates and allow them to allocate the available organs more successfully.

Positron emission tomography (PET) imaging has been evaluated and used as a noninvasive tool to assess graft function during an episode of AR, especially in the context of heart and lung transplantation. Tian et al. investigated the use of ML-based radiomics analysis of 18F-fluorodeoxyglucose PET images for monitoring allograft rejection in a rat lung transplant model (52). The researchers found that both the maximum standardized uptake value and radiomics score were correlated with histopathological criteria for rejection. ML models outperformed logistic regression models in detecting allograft rejection, with the optimal model achieving an AUC of 0.982. The study suggests that ML-based PET radiomics can enhance the monitoring of allograft rejection in lung transplantation.

Jen et al. explored the use of ML algorithms to predict DGF in renal transplant patients (53). The authors developed and optimized over 400,000 models based on donor data, with the best-performing models being neural network algorithms. These models showed comparable performance to logistic regression models. The study suggests that ML can improve outcomes in renal transplantation. Additionally, the development of automated ML pipelines, such as MILO (Machine Learning for Organ Transplantation), can facilitate the analysis of transplant data and the generation of prediction models. Zhou et al. presented a study that used the LASSO algorithm to identify protein biomarkers associated with the survival of renal grafts after transplantation (54). The study analyzed data from 47 kidney transplant patients and identified two proteins, KIM-1 and VEGF-R2, as significant predictors for graft loss. The authors also introduce a post-selection inference method to assess the statistical significance of the selected proteins. They addressed the issue of statistical significance in the selected predictors by proposing a post-selection inference method for the Cox regression model. This method provided a theoretically justified approach to reporting statistical significance by providing both confidence intervals and *p*-values for the selected predictors. This approach allowed the authors to establish pointwise statistical inference for each parameter in the Cox model and to guard against false discoveries of “weak signals” and model overfitting resulting from the LASSO regularized Cox regression. The proposed post-selection inference method was used to determine the statistical significance of the predictors KIM-1 and VEGF-R2 in predicting the hazard of allograft loss after renal transplantation. Yoo et al. analyzed data from a multicenter cohort of 3,117 patients and compared the power of ML models to conventional models in predicting graft survival in kidney transplant recipients (55). The ML methods, particularly survival decision tree models, outperformed conventional models in predicting graft survival. The authors also identified early AR as a significant risk factor for graft failure and highlighted the feasibility of using ML approaches in transplant medicine. Using ML methods, such as survival decision tree, bagging, RF, and ridge and lasso, increased the predictive power of graft survival compared to conventional models like decision tree and Cox regression. Specifically, the survival decision tree model increased the concordance index (C-index) to 0.80, with the episode of AR during the first-year post-transplant being associated with a 4.27-fold increase in the risk of graft failure. Additionally, survival tree modeling had higher predictive power than existing conventional decision tree models, increasing the reliability of identified factors in predicting clinical outcomes.

Cardiac allograft rejection remains a significant barrier to long-term survival for heart transplant recipients. The invasive and costly nature of endomyocardial biopsy, the gold standard for screening heart rejection, has prompted the exploration of non-invasive alternatives. Tong et al. used ML, particularly deep neural networks (DNNs), to offer a promising approach for such a solution (56). In this study, authors developed and evaluated a DNN model capable of predicting heart rejection using histopathological WSI as input. Trained on a dataset of WSIs from patients with and without heart rejection, the DNN model achieved an accuracy of 90% when evaluated on an independent dataset. Additionally, incorporating dropout, a regularization technique, further enhanced the model's performance, yielding an accuracy of 92%. These findings underlined the potential of DNNs as a non-invasive and cost-effective tool for predicting heart rejection, offering a promising alternative to endomyocardial biopsy. Further research should focus on developing DNN models capable of real-time prediction, enabling clinicians to make timely and informed treatment decisions.

Lau et al. used ML algorithms to predict graft failure after liver transplantation (57). They developed an algorithm using donor, recipient, and transplant factors and found that it had high accuracy in predicting graft failure. The study also compared the predictive ability of ML algorithms with traditional methods and highlighted the advantages of using RF classifiers. The ML algorithm, particularly the RF classifier, showed significant improvements in predicting transplant outcomes compared to traditional methods. The average AUC of the RF algorithm was 0.818, and 0.835 with artificial neural networks, indicating high predictive accuracy. In contrast, using the variables used to calculate the Donor Risk Index plus MELD score resulted in an AUC of 0.764, suggesting that the ML algorithm outperformed the traditional risk assessment methods. This demonstrates the potential of ML algorithms to provide more accurate predictions of transplant outcomes compared to conventional risk assessment tools. Table 4 summarizes all studies reviewed in this section. Code availability for all reviewed studies is listed in Table 5 when applicable.

**Table 4:** Summary of studies involving AI in post-transplantation prognostication.

Authors	Year	Application	Test	Comparison	Performance	Limitations	Data availability
Mulugeta et al. (40)	2023	Prediction of kidney graft failure post transplantation	Merit-based selected probabilistic models (Logistic regression, naïve Bayes, and artificial neural network) and tree-based ensemble (RF,bagged tree, and stochastic gradient boosting)		Bagged tree and random forest have best and equal discrimination performance with an AUC of 0.84 RF has the best calibration performance with a brier score of 0.045	Small sample size and minimum event per data	Retrospective cohort of kidney transplant recipients at St. Paul's Hospital Millennium Medical College National Kidney Transplantation Center in Ethiopia
Tian et al. (41)	2023	Providing a personalized and accurate prediction for overall survival in patients after lung transplantation	Random Survival Forests (RSF)	Cox regression	Excellent prediction performance with an integrated AUC of 0.879 and an integrated Brier score of 0.13	Single center retrospective nature of the study Omission of some potentially influential variables and predictors form the model Testing the model included data from both pediatric and adult patients	Liver transplantation patients at Wuxi People's Hospital between January 2017 and December 2019
Chen et al. (42)	2023	Prediction of sepsis in liver transplant recipients within 7 days after transplantation	Seven prediction ML models were used: LR,SVM,RF,GBM,ADA,GNB, and MLP		RF performed the best at predicting s7 day post operative sepsis with an AUC of 0.731, accuracy of 71.6%, sensitivity of 62.1% and specificity of 76.1% in internal validation and an AUC of 0.755 in external validation	Single center Retrospective nature of the study Sepsis might be confused with postoperative graft dysfunction *% of septic patients were missed by the models, making the ML models rather a supporting tool rather than a diagnostic measure	Liver Transplant patients at the Third Affiliated Hospital of Sun Yat-sen University, registered in the China Organ Transplant Response Systems (www.cot.org.cn)
Rogers et al. (43)	2023	Enhancing patient -specific mortality prediction and determining covariate interaction using recipient and donor data	Recipient Survival After Orthotopic Liver Transplantation (ReSOLT) (survival tree algorithm)		Effectively stratifies patients into 20 subgroups with distinct survival probabilities Superior performance compared to traditional survival prediction models with an AUC of 0.742 and C-index of 0.728	Retrospective nature of the study Analysis spans over more than 20 years, making data lack homogeneity in reporting some of the variables Advances in hepatitis C treatment might be overlooked	The UNOS database
Ivanics et al. (44)	2023	Predicting 90-day post Ltx mortality	ElasticNet, LASSO, LightGBM and Ridge models		Ridge performed the best in individual and harmonized data sets.	Selection and misclassification bias since the cohort did not include patients that dropped off the waitlist	Canadian Organ Replacement Registry (CORR, Canada); National Health Service Blood and Transplantation (NHSBT, United Kingdom), and United Network for Organ Sharing (UNOS, United States)
Raymond et al. (45)	2023	Enhancing risk stratification for kidney transplant recipients by refined predictions of survival using clinical data	Bayesian joint models		Excellent predictive performance of 0.82–0.868	Donor risk factors are not included. Immunosuppression adherence is not evaluated	The Paris Transplant Group database
Thongprayoon et al. (51)	2022	Risk stratifying black kidney transplant recipients	Machine learning consensus clustering approach		4 distinct clustered were identified. Cluster 2 had the most favorable outcomes in terms of death censored graft failure, patient death and allograft rejection Higher risk of rejection was found in clusters 1 and 3	Incomplete and non-consistent data Allograft rejection rate might be underestimated due to attrition bias	OPTN/UNOS database
Gupta et al. (48)	2022	Predicting length of stay in the hospital post heart transplantation in pediatric patients	Stepwise logistic regression, Gradient boosting, and Random Forest		Stepwise logistic regression was not inferior to machine learning models (GB and RF) in predicting length of hospital stay in pediatric cardiac transplant recipients	Retrospective nature of the study Data uses only day 30 as the cut-off for post operative hospital stay Physician behaviors and institutional practices were not taken into consideration when developing the prediction model	PHTS cohort
Miller et al. (49)	2022	Predicting 1 year and 90 day all-cause mortality after Cardiac transplantation Predicting survival post heart transplantation	RF, XGBoost, and L2 regularised logistic regression (LR) For survival: Random Survival Forest (RSF), Survival XGBoost, and L2 regularised Cox regession (CR)		RF achieved the highest 1-year survival predictive power with an AUC of 0.893 LR achieved the highest 90-day predictive power with an AUC of 0.674	Retrospective nature of the study Data might be mislabeled and some of them are missing	United Network of Organ Sharing (UNOS) database
Nitski et al. (50)	2021	Predicting cause of death at 1 or 5 years post liver transplantation	Deep learning models	Logistic regression	Deep learning methods outperformed logistic regression, with the Transformer model achieving the highest AUC of 0.804 and 0.733 for 1-year and 5-year prediction respectively	Registry based study with a retrospective nature and omission and missing data biases issues with coding of the primary cause of death	all liver transplant recipients in the Scientific Registry of Transplant Recipients (SRTR) and University Health Network (UHN) databases.
Tian et al. (52)	2022	Monitoring allograft rejection in lung transplant recipient rats	Machine learning based radiomics	Logistic regression	Machine learning models performed better than logistic regression-based models in detecting allograft rejection with a median AUC of 0.921. RF model achieved the highest predictive performance with AUC of 0.982	Difficulty translating the findings to human research PET is not disease specific; the high uptake is reflective of cellular activity that can be due to infection or cancer	
Jen et al. (53)	2021	Predicting delayed kidney graft dysfunction	Machine learning pipeline	Logistic regression	Neural network algorithm performed the best at predicting delayed graft dysfunction with the highest AUC of 0.7595	Single center small sample size	All deceased donor kidney transplants with known recipient DGF status at The University of California from January 1, 2010, to December 31, 2018
Zhou et al. (54)	2017	Predicting renal allograft survival based on proteomic biomarkers	LASSO		KIM-1 and VEGF-R2 were identified as predictors of graft loss	Small sample size Selection basis due to demographic skewness of the data	Kidney transplantation patients at the University of Michigan Hospital from 1990 to 2010
Yoo et al. (55)	2017	Predicting renal allograft survival	Survival decision tree, bagging, random forest, and ridge and lasso	Decision tree and Cox regression	Survival decision tree has the highest predictive power	Selection bias due to underrepresentation of various ethnicity Missing data such as DSA Absence of external validation	Kidney transplant patients at three tertiary care hospitals in Korea, between 1997 and 2012
Tong et al. (56)	2017	Assessing cardiac allograft whole slide image histopathological for rejection	Deep neural network		DNN achieved 90% accuracy in predicting rejection. When regularization and dropout were considered, the performance of DNN was improved to reach an accuracy of 92%	Small sample size	Whole-slide images collected from patients undergone heart transplants at Children's Healthcare of Atlanta (CHOA)
Lau et al. (57)	2017	Predicting graft failure after LTx	Machine learning models	Traditional models: DRI, SOFT Score, and DRI ± MELD	ML model outperforms traditional model with an AUC of 0.818.	Small sample size The model was developed on an observational database	Liver Transplant Database from Austin Health, Melbourne, Australia, from January 1988 to October 2013

**Table 5:** List of code or app availability when mentioned in reviewed studies.

Authors	Year	Code/application availability
Korfiatis et al. (4)	2022	Code available at: https://github.com/potis/MedCorSeg
Ram et al. (5)	2023	Detailed code is available in the supplementary materials of the paper
Yi et al. (7)	2022	Code is available in supplementary material
Yao et al. (8)	2022	Code is described in supplementary material
Yoon et al. (17)	2018	Code details are described in the Method section and in supplementary materials
Garcia-Canadilla et al. (21)	2022	Codes available in the supplementary material
Yoo et al. (23)	2023	Code is available at: https://github.com/AdeelK93/collapsibleTree/
Yi et al. (24)	2022	Code available in Supplementary Methods
Vaulet et al. (25)	2022	RejectClass is available as an online application at: https://rejectionclass.eu.pythonanywhere.com.
Fang et al. (28)	2023	Detailed code was described in supplementary Materials
Baardwijk et al. (29)	2022	Codes are available at: https://github.com/ErasmusMC-Bioinformatics/KidneyRejectionClassifier
Azhie et al. (32)	2023	Code is available at: https://github.com/divya031090/Fibrosis_LSTM
Halloran et al. (33)	2020	Code details are available in supplementary materials
Chen et al. (36)	1999	Code details available in the Method section of the paper
Woillard et al. (38)	2021	Immunosuppressant Bayesian Dose Adjustment expert system: www.pharmaco.chu-limoges.fr/
Mulugeta et al. (40)	2023	No application Code is not available
Chen et al. (42)	2023	Code: https://github.com/scikit-learn/scikit-learn Application: ML-Base Calculator to Estimate Sepsis after Liver Transplantation (http://wb.aidcloud.cn/zssy/sepsis_web.html)
Trouchot et al. (45)	2023	Code is included with details in supplementary material No application is available
Thongprayoon et al. (51)	2022	Code is available in eMEthods in the supplementary material of this paper. No application is available
Gupta et al. (48)	2022	Code is available through: https://github.com/bcjaeger/length-of-stay
Miller et al. (49)	2022	Code is available on: https://github.com/HCVE/unos-ml
Nitski et al. (50)	2021	Code is available at https://github.com/bowang-lab/Transplant_Time_Series.
Zhou et al. (54)	2017	Code is available in the Method section of the paper and details are provided in the supplementary material.
Tong et al. (56)	2017	Code available in Method section

## Trends and future directions

The application of AI in solid organ transplantation has been gaining traction in recent years. In particular, the experimentation with the use of ML and its subfield of deep learning has generated promising results. As evident in this review, the reported studies show that AI can outperform and at worst perform as well as classical statistical approaches or humans in executing the tasks it's trained on. This is a very promising step forward in the integration of AI tools into the decision-making process in the assessment, diagnosis, and treatment of transplant patients. Since we have only begun to witness the first attempts of leveraging AI in improving transplant outcomes, it is expected that studies will train AI models on more tasks. In addition, AI scientists will be scaling up the training and deployment of these AI tools by collecting more data from transplant centers around the world. Prospective studies with concurrent external validation should follow suit which will test the reliability of the trained AI models. If good performance is still exhibited in a prospective setting, that would pave the way for adoption of these models in clinical practice. However, to fully adopt the transplant AI models, what will remain to be achieved is robustness (58). We simply define robustness here as the ability of the AI model to tackle or adjust to incoming data affected by an arising factor or intervention. Attaining this attribute will likely require a breakthrough in machine learning theory. Robustness is a major issue since in a real-world setting and in clinical practice -through time- unexpected factors can shift the distribution of the patient population characteristics. So far, there is no consensus on how to address this issue. Nevertheless, promising work in causal representation learning (59) is encouraging and raises hope that robustness will soon be achieved by AI models.

## Concluding remarks

With all the computational power it employs, AI can provide the best risk assessment tool for transplant candidates and an efficient pipeline for organ allocation. Its use in transplantation is not limited to the pre-operative period, as it can be used to develop models for graft outcome assessment. Histologic evaluation is the gold standard for a diagnosis of rejection. However, it suffers from inconsistency and non-generalizability due to the complexity of the Banff Score and the operator-dependent nature of the biopsy procedure and biopsy reading. AI might provide a more consistent model to assess graft pathology that uses histological findings and complements them with more stable molecular signatures. In addition, AI showed promising results when used to predict graft function, morbidities, and mortality post-transplantation. Harnessing the power of AI and integrating those computational models will help guide clinicians make more personalized decisions in taking care of transplantation patients.
